# Acute Symptomatic Seizures and Risk of Epilepsy in Autoimmune Encephalitis: A Retrospective Cohort Study

**DOI:** 10.3389/fimmu.2022.813174

**Published:** 2022-02-23

**Authors:** Rui Zhong, Xinyue Zhang, Qingling Chen, Mengmeng Li, Xin Guo, Weihong Lin

**Affiliations:** ^1^ Department of Neurology, First Hospital, Jilin University, Changchun, China; ^2^ Department of Hepatology, Second People’s Clinical College of Tianjin Medical University, Tianjin, China; ^3^ Department of Neurology, The First Affiliated Hospital of Xi’an Jiaotong University, Xi’an, China

**Keywords:** risk of epilepsy, autoimmune encephalitis, EEG abnormality, immunotherapy delay, acute symptomatic seizures, a larger number of ASMs

## Abstract

**Purpose:**

To investigate the clinical characteristics of acute symptomatic seizures and the predictors of the development of epilepsy in patients with anti-NMDAR, anti-LGI1, and anti-GABABR encephalitis.

**Methods:**

We retrospectively screened the medical records of 86 hospitalized patients with confirmed autoimmune encephalitis (AE). The clinical characteristics of acute symptomatic seizures were analyzed. The predictors of the development of epilepsy were investigated using logistic regression analysis.

**Results:**

A total of 86 patients with AE were finally included. Eighty-six percent of patients (n = 74) experienced acute symptomatic seizures, and 28.4% of patients developed epilepsy during follow-up. Abnormal EEG findings were more frequent in AE patients with acute symptomatic seizures. A greater number of anti-seizure medications (ASMs), abnormal EEG findings, and delayed immunotherapy were found to be independently associated with the development of epilepsy.

**Conclusion:**

Acute symptomatic seizures are a common manifestation in AE patients. During follow-up, 28.4% of AE patients developed epilepsy. The independent factors that predicted the development of epilepsy after the acute phase included a larger number of ASMs, EEG abnormalities, and delayed immunotherapy. In clinical practice, we should prioritize immunotherapy to control acute seizures as soon as possible. For AE patients with an increased risk of developing epilepsy, early withdrawal of ASM is not recommended.

## Introduction

It has been increasingly acknowledged in recent years that autoimmune encephalitis (AE) refers to a number of inflammatory autoimmune disorders of the brain that are associated with autoantibodies against neurosurface, synaptic or neuronal intracytoplasmic antigens (1–3). Acute symptomatic seizures are common clinical manifestations in the acute phase of AE, and more than half of AE patients experience seizure onset (4, 5). The effect of anti-seizure medications (ASMs) on acute seizure control is limited, and the responses to ASMs are poor in AE patients (6). Early withdrawal of ASM is not associated with the development of epilepsy after the acute phase (7). However, prior evidence has shown good acute seizure control outcomes in most AE patients (8, 9). Freedom from seizures is achieved soon after the initiation of immunotherapy in most AE patients with seizures (6).

Epilepsy is a common and serious brain disease, and an autoimmune response is a known etiology of epilepsy (5, 10). It has been reported that most AE patients treated with immunotherapy do not develop chronic epilepsy after the acute phase (6). However, the risk of developing epilepsy in AE patients varies (incidence ranging from 12.5% to 57.89%) (11, 12). To improve preventive strategies, it is necessary to identify AE patients who are at increased risk of developing epilepsy after the acute phase. Long-term ASM treatment should be recommended for such patients (13).

Studies that focused on the clinical features of acute symptomatic seizures and the predictors of epilepsy development in AE patients are limited, and their findings are controversial (4, 7, 11). Thus, we aimed to answer the following questions in this study: How many AE patients experience acute symptomatic seizures in the acute phase? Which demographic or clinical characteristics are associated with acute symptomatic seizures? How many patients develop epilepsy after the acute phase of AE? What are the predictors of the development of epilepsy in AE patients?

## Methods

### Participants

This retrospective observational study was based on hospitalized patients diagnosed with AE after the examination of cerebrospinal fluid (CSF) samples for anti-neural antibodies at the Department of Neurology, First Hospital of Jilin University, between September 2013 and November 2020. For each potential patient, antibodies were detected in CSF samples with cell-based assays rather than serum samples due to the better quality of the results. Newly diagnosed hospitalized patients with anti-NMDAR, anti-LGI1, and anti-GABABR encephalitis were consecutively enrolled in this study. AE was diagnosed and defined according to the diagnostic criteria for AE published in 2016 (14). We excluded patients who (1) had been diagnosed with epilepsy, stroke, and/or other brain diseases before the onset of AE that might influence acute seizure characteristics and outcomes; (2) had laboratory evidence of infectious encephalitis (e.g., viral, bacteria, mycobacterium tuberculosis, parasitic, or fungal encephalitis); (3) had other types of AE (not anti-NMDAR, anti-GABABR, or anti-LGI1 encephalitis) or coexisting antibodies; and (4) were not receiving immunotherapy treatment during the acute phase, died, or were lost to follow-up after discharge. The Ethics Committee of the First Hospital of Jilin University granted ethical approval for this retrospective observational study and waived the requirement for written informed consent. This study was carried out according to the Declaration of Helsinki.

### Data Collection

Demographic and clinical data were gathered retrospectively from electronic databases or by personal telephone interviews with patients and their relatives; data included sex, age at onset, clinical symptoms, tumor presence, acute seizure characteristics (e.g., onset with seizure, repeated acute seizures, status epilepticus, and type of seizure), CSF findings, 24-hour video electroencephalography (EEG) and brain magnetic resonance imaging (MRI) findings during the acute phase of AE (e.g., abnormal brain MRI findings and evidence of cortical involvement on brain MRI), intensive care unit (ICU) admission, immunotherapy treatment and seizure outcomes.

### Definitions

We defined the acute phase of AE as the first 3 months after the onset of AE symptoms. We used the term “acute symptomatic seizures” proposed by the International League Against Epilepsy (ILAE) to refer to seizures occurring in the acute phase of AE (5). The presence of epileptiform discharges (focal or generalized spike waves) on an EEG was defined as an abnormality. We employed the ILAE classification proposal and guidelines to define status epilepticus (15) and to classify the type of seizures (10, 16). A cutoff of 5 min of seizure duration was applied to define status epilepticus (15). The type of seizure was classified as focal, generalized or faciobrachial dystonic seizures (FBDS) based on clinical symptoms in accordance with the ILAE guidelines (10, 16). Epilepsy is defined as two unprovoked seizures occurring more than 24 h apart or a single unprovoked seizure with high recurrence risk (i.e., >60% over the next 10 years) (10). Delayed immunotherapy referred to immunotherapy that was started 28 days after disease onset (6). A CSF antibody titer ≤1:10 was scored as (+); >1:10 and ≤1:100 as (++); and >1:100 as (+++).

### Follow-Up

Patients with acute seizures were followed with telephone interviews and/or clinical visits for at least 6 months after the acute phase of AE. Formal follow-up seizure outcome assessments were performed every 6 months. Epileptic seizures after the acute phase of AE were investigated and confirmed with patients or their relatives at each follow-up point. Furthermore, patients were asked to visit their doctor within days of experiencing an epileptic seizure or possible encephalitis relapse. Epileptic seizures were distinguished from encephalitis relapse by clinicians *via* a face-to-face interview. Patients who experienced one or more epileptic seizures after the acute phase of AE were considered to have epilepsy. The consultation, diagnosis, and treatment of epilepsy were based on physician experience.

### Statistical Analysis

All data analyses were performed using SPSS version 26.0. Continuous variables are presented as medians (interquartile ranges [IQRs]), and categorical variables are presented as counts (percentages). Comparisons between multiple groups were performed with the Kruskal–Wallis test for continuous variables. We compared differences between the two groups using the Mann–Whitney U test for continuous variables. Pearson’s chi-squared or Fisher’s exact test was employed for comparisons of categorical variables. If multiple variables were found to be associated with seizure outcome in the univariate analyses (p<0.05), stepwise logistic regression analysis was performed to investigate the independent predictors of seizure outcome. The results are expressed as adjusted odds ratios (ORs) with corresponding 95% confidence intervals (CIs). Two-sided P values < 0.05 were considered statistically significant.

## Results

### Patient Characteristics

**Figure 1:**
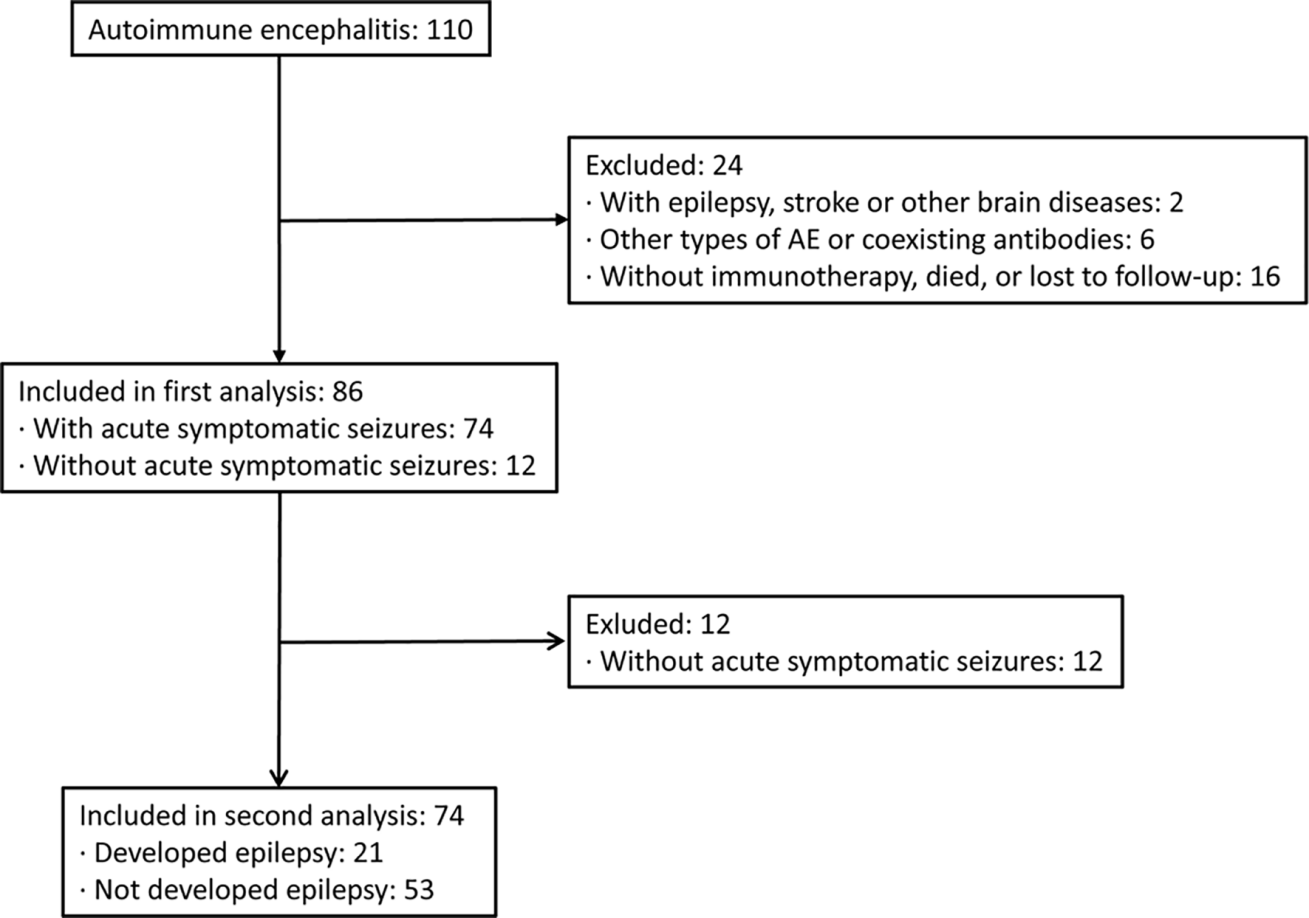
Flow diagram of patient inclusion and grouping.

We screened the medical records of 110 hospitalized patients with confirmed AEs between September 2013 and November 2020 in our center. Twenty-four patients were excluded based on the exclusion criteria. A total of 86 patients with AE were included in the analysis, with a median age at onset of 48 years and a male percentage of 44% (**Figure 1**). In this cohort, 37 patients were positive for anti-NMDAR (43%), 34 patients (40%) for anti-LGI1, and 15 patients for anti-GABABR (17%) encephalitis. Among the cases, 86% of patients (n = 74) experienced acute symptomatic seizures in the acute phase of AE, while 14% did not report acute seizures. The demographic and clinical characteristics of these patients are shown in **Table 1**. Patients with NMDAR antibodies were younger, and patients with anti-GABABR antibody were more likely to require admission to the ICU. Additionally, there were significant differences in the rates of fever, movement disorders, abnormal brain MRI findings, administration of human chorionic gonadotrophin (HCG), and immunotherapy delay among patients in the three groups.

**Table 1:** Patient characteristics by type of AE.

Characteristics	Total (n=86)	NMDAR (n=37)	LGI1 (n=34)	GABABR (n=15)	P-value
Male	44 (51.2)	20 (54.1)	17 (50.0)	7 (46.7)	0.877
Age at onset, (Years)	48 (31, 60)	30 (21, 41)	58 (48, 67)	57 (52, 61)	<0.001
Acute symptomatic seizure	74 (86.0)	30 (81.1)	30 (88.2)	14 (93.3)	0.543
Fever (>37.5°C)	29 (33.7)	19 (51.4)	3 (8.8)	7 (46.7)	<0.001
Psychiatric symptoms	54 (62.8)	26 (70.3)	19 (55.9)	9 (60.0)	0.443
Movement disorder	38 (44.2)	23 (62.2)	13 (38.1)	2 (13.3)	0.004
Cognitive impairment	59 (68.6)	23 (62.2)	28 (82.4)	8 (53.3)	0.066
Dysarthria	19 (22.1)	12 (32.4)	4 (11.8)	3 (20.0)	0.101
Tumor presence	9 (10.5)	5 (13.5)	2 (5.9)	2 (13.3)	0.574
EEG (Abnormal)	44 (51.2)	14 (37.8)	19 (55.9)	11 (73.3)	0.053
Brain MRI (Abnormal)	48 (55.8)	19 (51.4)	24 (70.6)	5 (33.3)	0.041
CSF analysis (Abnormal)	74 (86.0)	33 (89.2)	26 (76.5)	15 (100.0)	0.075
Antibody titer					
+	31 (36.0)	15 (40.5)	12 (35.3)	4 (26.7)	0.816
++	40 (46.5)	17 (45.9)	16 (47.1)	7 (46.7)	
+++	15 (17.4)	5 (13.5)	6 (17.6)	4 (26.7)	
Administration of HGG	66 (76.7)	34 (91.9)	22 (64.7)	10 (66.7)	0.01
Administration of corticosteroids	76 (88.4)	34 (91.9)	29 (85.3)	13 (86.7)	0.667
Immunotherapy delay	38 (44.2)	10 (27.0)	23 (67.6)	5 (33.3)	0.002
ICU admission	36 (41.9)	23 (62.2)	4 (11.8)	9 (60.0)	<0.001

AE, autoimmune encephalitis; NMDAR, N-methyl-D-aspartate receptor; LGI1, leucin-rich glioma inactivated-1; GABABR, g-aminobutyric acid type B receptor; CSF, cerebrospinal fluid; EEG, electroencephalogram; MRI, magnetic resonance imaging; HGG, human gamma globulin; ICU, intensive care unit.

+: antibody titer ≤1:10; ++: antibody titer >1:10 and ≤1:100; +++: antibody titer >1:100 as (+++).

### Seizure Characteristics

The seizure characteristics of AE patients are summarized in **Table 2**. Of the 74 patients with acute symptomatic seizures, 64.9% reported onset with seizure, and 36.5% experienced status epilepticus. Compared with those in the other two groups, patients in the GABABR group were more likely to experience status epilepticus (p=0.01), and patients in the LGI1 group were more prone to abnormal brain MRI results (p=0.049). FBDS is a unique seizure type only reported by patients with LGI1 antibodies, and the type of seizures differed significantly among the 3 groups (p<0.001). Approximately half of the patients were treated with 1 ASM, and 17.6% received 3 or more ASMs. The median follow-up period after discharge was 21 months (IQR 8–30, range 6–60 months). During follow-up, 28.4% of AE patients experienced at least one epileptic seizure and developed epilepsy.

**Table 2:** Seizure characteristics by type of AE.

Seizure characteristics	Total (n=74)	NMDAR (n=30)	LGI1 (n=30)	GABABR (n=14)	P-value
Onset with seizure	48 (64.9)	15 (50.0)	21 (70.0)	12 (85.7)	0.061
Repeated acute seizures	68 (91.9)	26 (86.7)	29 (96.7)	13 (92.9)	0.42
Status epilepticus	27 (36.5)	8 (26.7)	9 (30.0)	10 (71.4)	0.01
Type of seizures					
FBDS	15 (20.3)	0 (0)	15 (50.0)	0 (0)	<0.001
Generalized seizure	31 (41.9)	14 (46.7)	8 (26.7)	9 (64.3)	
Focal seizure	18 (37.8)	16 (53.3)	7 (23.3)	5 (35.7)	
Number of ASMs					
1	39 (52.7)	15 (50.0)	17 (56.7)	7 (50.0)	0.509
2	22 (29.7)	7 (23.3)	10 (33.3)	5 (35.7)	
≥3	13 (17.6)	8 (26.7)	3 (4.5)	2 (14.2)	
EEG (Abnormal)	42 (56.8)	14 (46.7)	17 (56.7)	11 (78.6)	0.138
Epilepsy after acute phase	21 (28.4)	7 (23.3)	8 (26.7)	6 (42.9)	0.445

AE, autoimmune encephalitis; NMDAR, N-methyl-D-aspartate receptor; LGI1, leucin-rich glioma inactivated-1; GABABR, g-aminobutyric acid type B receptor; FBDS, faciobrachial dystonic seizure; ASMs, antiseizure medications; EEG, electroencephalogram.

### Factors Associated With Acute Symptomatic Seizures in All AE Patients

A total of 86.1% of AE patients experienced acute symptomatic seizures. The demographic and clinical characteristics of patients with and without acute symptomatic seizures are described in **Table 3**. Abnormal EEG findings were more frequent in AE patients with acute symptomatic seizures (p=0.01). No other factor was found to be associated with an increased likelihood of acute seizures in AE patients.

**Table 3:** Univariate analysis of factors associated with acute symptomatic seizures in total AE patients.

Variables	Acute symptomatic seizure in acute phase		P-value
	Yes (n=74)	No (n=12)	
Male	37 (50.0)	7 (58.3)	0.592
Age at onset, (Years)	48 (31, 59)	50 (32, 71)	0.239
Fever (>37.5°C)	22 (29.7)	7 (58.3)	0.106
Psychiatric symptoms	48 (64.9)	6 (50.0)	0.505
Movement disorder	31 (41.9)	7 (58.3)	0.287
Cognitive impairment	50 (67.6)	9 (75.0)	0.858
Dysarthria	16 (21.6)	3 (25.0)	1
Tumor presence	9 (12.0)	0 (0)	0.442
EEG (Abnormal)	42 (56.8)	2 (16.7)	0.01
Brain MRI (Abnormal)	43 (58.1)	5 (41.7)	0.287
CSF analysis (Abnormal)	64 (86.5)	10 (83.3)	1
Antibodies			
NMDAR	30 (40.5)	7 (58.3)	0.459
LGI1	30 (40.5)	4 (33.3)	
GABABR	14 (18.9)	1 (8.3)	
Antibody titer			
+	28 (37.8)	3 (25.0)	0.639
++	33 (44.6)	7 (58.3)	
+++	13 (17.6)	2 (16.7)	
Administration of HGG	57 (77.0)	9 (75.0)	1
Administration of corticosteroids	67 (90.5)	9 (75.0)	0.284
Immunotherapy delay	34 (45.9)	4 (33.3)	0.414
ICU admission	32 (43.2)	4 (33.3)	0.519

NMDAR, N-methyl-D-aspartate receptor; LGI1, leucin-rich glioma inactivated-1; GABABR, g-aminobutyric acid type B receptor; CSF, cerebrospinal fluid; EEG, electroencephalogram; MRI, magnetic resonance imaging; HGG, human gamma globulin; ICU, intensive care unit.

+: antibody titer ≤1:10; ++: antibody titer >1:10 and ≤1:100; +++: antibody titer >1:100 as (+++).

### Factors Associated With Acute Symptomatic Seizures in Patients With Anti-NMDAR, Anti-LGI1, and Anti-GABABR Encephalitis

Among the patients with anti-NMDAR encephalitis, individuals with abnormal EEG findings were more likely to experience acute symptomatic seizures than those with normal EEG findings. No other significant associations were found between acute seizures and potentially related variables. Among the patients with anti-LGI1 encephalitis, no factor was found to be associated with an increased risk of acute symptomatic seizures. Similarly, there was no significant association between acute symptomatic seizures and the potentially related variables among patients with anti-GABABR encephalitis. For details, see **Table 4**.

**Table 4:** Univariate analysis of factors associated with acute symptomatic seizures in patients with anti-NMDAR, anti-LGI1, and anti-GABABR encephalitis.

Variables	NMDAR (n=37)		P-value	LGI1 (n=34)		P-value	GABABR (n=15)		P-value
	Acute symptomatic seizure			Acute symptomatic seizure			Acute symptomatic seizure		
	Yes (n=30)	No (n=7)		Yes (n=30)	No (n=4)		Yes (n=14)	No (n=1)	
Male	16 (53.3)	4 (57.1)	1	15 (50.0)	2 (50.0)	1	6 (42.9)	1 (100.0)	0.467
Age at onset, (Years)	30 (20, 38)	37 (28, 59)	0.149	58 (48, 66)	61 (50, 74)	0.519	56 (52, 60)	79	_
Fever (>37.5°C)	14 (46.7)	5 (71.4)	0.403	2 (6.7)	1 (25.0)	0.322	6 (42.9)	1 (100.0)	0.467
Psychiatric symptoms	22 (73.3)	4 (57.1)	0.7	17 (56.7)	2 (50.0)	1	9 (64.3)	0	0.4
Movement disorder	18 (60.0)	5 (71.4)	0.687	12 (40.0)	1 (25.0)	1	1 (7.1)	1 (100.0)	0.133
Cognitive impairment	19 (63.3)	4 (57.1)	1	24 (80.0)	4 (100.0)	1	7 (50.0)	1 (100.0)	1
Dysarthria	10 (33.3)	2 (28.6)	1	4 (13.3)	0	1	2 (14.3)	1 (100.0)	0.2
Tumor presence	5 (16.7)	0	0.56	2 (6.7)	0	1	2 (14.3)	0	1
EEG (Abnormal)	14 (46.7)	0	0.031	17 (56.7)	2 (50.0)	1	11 (78.6)	0	0.267
Brain MRI (Abnormal)	16 (53.3)	3 (42.9)	0.693	22 (73.3)	2 (50.0)	0.564	5 (35.7)	0	1
Cortical involvement in brain MRI	10 (33.3)	3 (42.9)	0.678	20 (66.7)	2 (50.0)	0.602	5 (35.7)	0	1
CSF analysis (Abnormal)	27 (90.0)	6 (85.7)	1	23 (76.7)	3 (75.0)	1	14 (100.0)	1 (100.0)	_
**Antibody titer**									
+	13 (43.3)	2 (28.6)	0.762	11 (36.7)	1 (25.0)	0.43	4 (28.6)	0	0.229
++	13 (43.3)	4 (57.1)		13 (43.3)	3 (75.0)		7 (50.0)	0	
+++	4 (13.3)	1 (14.3)		6 (20.0)	0		3 (21.4)	1 (100.0)	
Administration of HGG	29 (96.7)	5 (71.4)	0.086	19 (63.3)	3 (75.0)	1	9 (64.3)	1 (100.0)	1
Administration of corticosteroids	28 (93.3)	6 (85.7)	0.477	26 (86.7)	3 (75.0)	0.488	13 (92.9)	0	0.133
Immunotherapy delay	8 (26.7)	2 (28.6)	1	21 (70.0)	2 (50.0)	0.58	5 (35.7)	1 (100.0)	1
ICU admission	20 (66.7)	3 (42.9)	0.39	3 (10.0)	1 (25.0)	0.409	9 (64.3)	0	0.4

NMDAR, N-methyl-D-aspartate receptor; LGI1, leucin-rich glioma inactivated-1; GABABR, g-aminobutyric acid type B receptor; CSF, cerebrospinal fluid; EEG, electroencephalogram; MRI, magnetic resonance imaging; HGG, human gamma globulin; ICU, intensive care unit.

+: antibody titer ≤1:10; ++: antibody titer >1:10 and ≤1:100; +++: antibody titer >1:100 as (+++).

### Factors Associated With the Development of Epilepsy in All AE Patients

During follow-up, 28.4% of AE patients developed epilepsy. A univariate analysis was carried out to investigate the factors associated with the development of epilepsy. The demographic and clinical characteristics of patients with and without epilepsy after the acute phase are presented in **Table 5**. The occurrence of status epilepticus (p=0.02), a larger number of ASMs (p=0.032), and abnormal EEG findings (p=0.034) were found to have significant associations with the development of epilepsy after the acute phase in all AE patients. Furthermore, AE patients with immunotherapy delay were more prone to developing epilepsy (p=0.006). In addition, age at onset, brain MRI abnormality, type of AE, antibody titers, and ICU admission were not significantly different between the groups of AE patients with and without the development of epilepsy (p>0.05).

**Table 5:** Univariate analysis of factors associated with the development of epilepsy in total AE patients.

Variables	Epilepsy after acute phase		P-value
	Yes (n=21)	No (n=53)	
Male	12 (57.1)	25 (47.2)	0.439
Age at onset, (Years)	52 (34, 58)	47 (30, 60)	0.853
Onset with seizure	15 (71.4)	33 (62.3)	0.457
Repeated acute seizures	21 (100.0)	47 (88.7)	0.256
Status epilepticus	12 (57.1)	15 (28.3)	0.02
Type of seizures			
FBDS	6 (28.6)	9 (17.0)	0.534
Generalized seizure	8 (38.1)	23 (43.4)	
Focal seizure	7 (33.3)	21 (39.6)	
Number of ASMs	2 (1, 3)	1 (1, 2)	0.032
Fever (>37.5°C)	7 (33.3)	15 (28.3)	0.669
Psychiatric symptoms	12 (57.1)	36 (67.9)	0.381
Movement disorder	8 (38.1)	23 (43.4)	0.677
Cognitive impairment	16 (76.2)	34 (64.2)	0.319
Dysarthria	5 (23.8)	11 (20.8)	1
Tumor presence	3 (14.3)	6 (11.3)	1
EEG (Abnormal)	16 (76.2)	26 (49.1)	0.034
Brain MRI (Abnormal)	13 (61.9)	30 (56.6)	0.677
CSF analysis (Abnormal)	18 (85.7)	46 (86.8)	1
Antibodies			
NMDAR	7 (33.3)	23 (43.4)	0.394
LGI1	8 (38.1)	22 (41.5)	
GABABR	6 (28.6)	8 (15.1)	
Antibody titer			
+	6 (28.6)	22 (41.5)	0.391
++	12 (57.1)	21 (39.6)	
+++	3 (14.3)	10 (18.9)	
Administration of HGG	17 (81.0)	40 (75.5)	0.842
Administration of corticosteroids	20 (95.2)	47 (88.7)	0.668
Immunotherapy delay	15 (71.4)	19 (35.8)	0.006
ICU admission	11 (52.4)	21 (39.6)	0.318

NMDAR, N-methyl-D-aspartate receptor; LGI1, leucin-rich glioma inactivated-1; GABABR, g-aminobutyric acid type B receptor; FBDS, faciobrachial dystonic seizure; ASMs, antiseizure medications; CSF, cerebrospinal fluid; EEG, electroencephalogram; MRI, magnetic resonance imaging; HGG, human gamma globulin; ICU, intensive care unit.

+: antibody titer ≤1:10; ++: antibody titer >1:10 and ≤1:100; +++: antibody titer >1:100 as (+++).

### Factors Associated With the Development of Epilepsy in Patients With Anti-NMDAR, Anti-LGI1, and Anti-GABABR Encephalitis

Subgroup analyses were performed to identify factors associated with the development of epilepsy according to the type of AE. Among patients with anti-NMDAR encephalitis, no factor was found to be associated with the development of epilepsy after the acute phase. Among patients with anti-LGI1 encephalitis, there were significant associations of the development of epilepsy with the occurrence of status epilepticus (p=0.032) and a larger number of ASMs (p=0.05). Patients with abnormal EEG findings (p=0.092) and immunotherapy delay (p=0.067) were prone to developing epilepsy. However, the differences did not reach statistical significance. Among patients with anti-GABABR encephalitis, age at onset (p=0.013) was significantly different between the groups with and without the development of epilepsy. Immunotherapy delay (p=0.091) tended to be associated with the development of epilepsy, but the association was not statistically significant. For details, see **Table 6**.

**Table 6:** Univariate analysis of factors associated with the development of epilepsy in patients with anti-NMDAR, anti-LGI1, and anti-GABABR encephalitis.

Variables	NMDAR (n=30)		P-value	LGI1 (n=30)		P-value	GABABR (n=14)		P-value
	Epilepsy			Epilepsy			Epilepsy		
	Yes (n=7)	No (n=23)		Yes (n=8)	No (n=22)		Yes (n=6)	No (n=8)	
Male	4 (57.1)	12 (52.2)	1	4 (50.0)	11 (50.0)	1	4 (66.7)	2 (25.0)	0.277
Age at onset, (Years)	34 (26, 41)	29 (21, 37)	0.36	60 (52, 69)	58 (48, 66)	0.534	53 (28, 56)	59 (57, 62)	0.013
Onset with seizure	3 (42.9)	12 (52.2)	1	7 (87.5)	14 (63.6)	0.374	5 (83.3)	7 (87.5)	1
Repeated acute seizures	7 (100.0)	19 (82.6)	0.548	8 (100.0)	21 (95.5)	1	6 (100.0)	7 (87.5)	1
Status epilepticus	2 (28.6)	6 (26.1)	1	5 (62.5)	4 (18.2)	0.032	5 (83.3)	5 (62.5)	0.58
**Type of seizures**									
FBDS	_	_	0.675	6 (75.0)	9 (40.9)	0.117	_	_	1
Generalized seizure	4 (57.1)	10 (43.5)		0	8 (36.4)		4 (66.7)	5 (62.5)	
Focal seizure	3 (42.9)	13 (56.5)		2 (25.0)	5 (22.7)		2 (33.3)	3 (37.5)	
Number of ASMs	2 (1, 3)	1 (1, 3)	0.774	2 (2, 3)	1 (1, 2)	0.05	1 (1, 2)	2 (1, 2)	0.414
Fever (>37.5°C)	4 (57.1)	10 (43.5)	0.675	0	2 (9.1)	1	3 (50.0)	3 (37.5)	1
Psychiatric symptoms	4 (57.1)	18 (78.3)	0.345	5 (62.5)	12 (54.5)	1	3 (50.0)	6 (75.0)	0.58
Movement disorder	5 (71.4)	13 (56.5)	0.669	3 (37.5)	9 (40.9)	1	0	1 (12.5)	1
Cognitive impairment	5 (71.4)	14 (60.9)	1	8 (100.0)	16 (72.7)	0.155	3 (50.0)	4 (50.0)	1
Dysarthria	2 (28.6)	8 (34.8)	1	2 (25.0)	2 (9.1)	0.284	1 (16.7)	1 (12.5)	1
Tumor presence	1 (14.3)	4 (17.4)	1	1 (12.5)	1 (4.5)	0.469	1 (16.7)	1 (12.5)	1
EEG (Abnormal)	4 (57.1)	10 (43.5)	0.675	7 (87.5)	10 (45.5)	0.092	5 (83.3)	6 (75.0)	1
Brain MRI (Abnormal)	4 (57.1)	12 (52.2)	1	7 (87.5)	15 (68.2)	0.391	2 (33.3)	3 (37.5)	1
Cortical involvement in brain MRI	4 (57.1)	6 (26.1)	0.181	7 (87.5)	13 (59.1)	0.21	2 (33.3)	3 (37.5)	1
CSF analysis (Abnormal)	7 (100.0)	20 (87.0)	1	5 (62.5)	18 (81.8)	0.345	6 (100.0)	8 (100.0)	_
**Antibody titer**									
+	3 (42.9)	10 (43.5)	0.445	2 (25.0)	9 (40.9)	0.721	1 (16.7)	3 (37.5)	0.545
++	4 (57.1)	9 (39.1)		4 (50.0)	9 (40.9)		4 (66.7)	3 (37.5)	
+++	0	4 (17.4)		2 (25.0)	4 (18.2)		1 (16.7)	2 (25.0)	
Administration of HGG	7 (100.0)	22 (95.7)	1	5 (62.5)	14 (63.6)	1	5 (83.3)	4 (50.0)	0.301
Administration of corticosteroids	7 (100.0)	21 (91.3)	1	7 (87.5)	19 (86.4)	1	6 (100.0)	7 (87.5)	1
Immunotherapy delay	3 (42.9)	5 (21.7)	0.345	8 (100.0)	13 (59.1)	0.067	4 (66.7)	1 (12.5)	0.091
ICU admission	5 (71.4)	15 (65.2)	1	2 (25.0)	1 (4.5)	0.166	4 (66.7)	5 (62.5)	1

NMDAR, N-methyl-D-aspartate receptor; LGI1, leucin-rich glioma inactivated-1; GABABR, g-aminobutyric acid type B receptor; FBDS, faciobrachial dystonic seizure; ASMs, antiseizure medications; CSF, cerebrospinal fluid; EEG, electroencephalogram; MRI, magnetic resonance imaging; HGG, human gamma globulin; ICU, intensive care unit.

+: antibody titer ≤1:10; ++: antibody titer >1:10 and ≤1:100; +++: antibody titer >1:100 as (+++).

### Independent Factors Associated With Seizure Outcomes in All AE Patients

To identify the independent factors associated with the development of epilepsy in all AE patients, variables with a p value <0.05 in the univariate analyses were included in the multivariable logistic regression analysis. In the stepwise logistic analysis adjusted for the occurrence of status epilepticus, the number of ASMs, abnormal EEG findings, and immunotherapy delay, the multivariate results were largely consistent with those from the univariate analyses. A larger number of ASMs (adjusted OR 2.548, 95% CI 1.235–5.255; p = 0.011) was independently associated with an increased risk of the development of epilepsy after the acute phase of AE. Compared with patients with normal EEG results, those with EEG abnormalities had 3.919-fold higher odds of developing epilepsy (adjusted OR 3.919, 95% CI 1.098–13.992; p = 0.035). Patients with immunotherapy delay had 6.432-fold higher odds of developing epilepsy (adjusted OR 6.432, 95% CI 1.825–22.662; p = 0.004). For details, see **Table 7**.

**Table 7:** Multivariable logistic regression analysis of factors independently associated with the development of epilepsy in total AE patients.

Variables	Adjusted OR	95% CI	P-value
Number of ASMs	2.548	1.235-5.255	0.011
EEG (Abnormal)	3.919	1.098-13.992	0.035
Immunotherapy delay	6.432	1.825-22.662	0.004

ASMs, antiseizure medications; EEG, electroencephalogram; OR, odds ratio; CI, confidence interval.

## Discussion

This retrospective, observational study evaluated the clinical features of acute symptomatic seizures and the predictors of the development of chronic epilepsy in patients with anti-LGI1, anti-NMDAR, and anti-GABABR encephalitis. This study showed that 86.0% of AE patients presented with acute symptomatic seizures in the acute phase. Abnormal EEG results were associated with an increased risk of acute symptomatic seizures. During a median follow-up of 21 months, 28.4% of patients with acute seizures developed epilepsy. The independent factors that predicted the development of epilepsy after the acute phase included a larger number of ASMs, EEG abnormalities, and immunotherapy delay. The occurrence of status epilepticus in the acute phase was associated with the development of epilepsy. However, this association was not significant in the multivariable logistic regression analysis.

Seizures are a common manifestation of AE in patients in the acute phase. It is important to distinguish the term “acute symptomatic seizures” from the term “autoimmune-associated epilepsy”, which has clinical and therapeutic implications. According to the definitions of the ILAE, the term “acute symptomatic seizures” refers to seizures occurring in the acute phase of AE (5). The term “autoimmune-associated epilepsy” is defined as chronic seizures attributed to secondary brain diseases (5). It has been reported that patients with anti-GABABR encephalitis are more likely to experience acute symptomatic seizures than are those with anti-NMDAR encephalitis (6, 17). These trends can be observed in our results, despite the nonsignificant differences. This may be due to the relatively small sample size in our study. We also found that the occurrence of status epilepticus was more frequent in the group with anti-GABABR encephalitis than in the other two groups. Marienke et al. reported similar findings that patients with anti-GABABR encephalitis were more likely to experience status epilepticus, and 68% of them even had refractory status epilepticus (6). A significant difference was observed in terms of seizure type among groups with anti-NMDAR, anti-LGI1 and anti-GABABR encephalitis, which was in line with the results of prior literature (4, 6). FBDS is a unique type of seizure and was only observed in patients with anti-LGI1 encephalitis, which was more frequently reported than other seizure types (18, 19).

In this study, we found that 86% of AE patients experienced acute symptomatic seizures in the acute phase. In a prior cohort of patients with anti-NMDAR encephalitis from West China, Hong and colleagues reported that 80.7% of patients experienced acute seizures, which was similar to our reported incidence (8). Additionally, it is not surprising that EEG abnormalities were associated with an increased likelihood of seizures in AE patients in the acute phase. However, in the subgroup analysis according to the type of AE, this association was significant only in patients with anti-NMDAR encephalitis. This result may be partly explained by the reduced sample size in the subgroup analysis. Prior evidence suggests that epileptiform activity as captured electrographically is most common at the peak of illness, and approximately 1/3 of these AE patients present with status epilepticus (20).

In this study, we showed that during a median follow-up of 21 months, 28.4% of AE patients with acute seizures developed epilepsy. This result is similar to the results of Ding et al., who reported that 21.3% of AE patients developed epilepsy (7). Wang and colleagues reported that 51.78% of AE patients did not achieve seizure-free status after the acute phase (4). In another recent study from Spain, 57.89% of patients developed epilepsy after AE (11). One possible explanation for the varying reported incidence in AE patients could be the different sample characteristics among studies.

We discovered that a larger number of ASMs was independently associated with an increased risk of the development of epilepsy in AE patients. In the subgroup analysis, this association was significant only in patients with anti-LGI1 encephalitis. ASM was prescribed to control seizures in the acute phase, and a larger number of ASMs was related to increased seizure severity and worse autoimmune processes in the acute phase, which may contribute to the development of epilepsy after the acute phase. It was recently reported that patients with epilepsy who had a history of status epilepticus were more likely to receive a larger number of ASMs than those without a history of status epilepticus (21). Thus, it is not surprising that in AE patients, a larger number of ASMs may be related to the occurrence of status epilepticus, which was also identified as a risk factor for the development of epilepsy in the univariate analysis. However, the association between the occurrence of status epilepticus at the acute phase and the development of epilepsy was not significant in the multivariable logistic regression analysis. This may be due to the interaction between the number of ASMs and the occurrence of status epilepticus in AE patients. There is evidence that the effects of ASMs on seizure control are limited, and the use of immunotherapy is more effective in achieving seizure freedom in AE patients (6). Additionally, prior literature has suggested that early withdrawal of ASMs was not associated with the development of epilepsy in patients (7). EEG abnormalities were identified as another independent risk factor for the development of epilepsy in AE patients. However, the relationship was not significant in the subgroup analysis, which may be due to the smaller sample size. A significant relationship between abnormal EEG results and the development of epilepsy was also reported by other researchers (7). Lu et al. proposed that EEG abnormalities evaluated by the grand total EEG score may be used as a predictor of long-term seizure outcomes associated with autoimmune encephalitis (22). However, previous studies by Hong et al. (8) and Zhang et al. (4) failed to observe the predictive role of abnormal EEG results in unfavorable seizure outcomes. Differences in the time points of EEG examinations may lead to inconsistent findings among studies. Our study also indicated that immunotherapy delay in the acute phase contributed to the development of epilepsy in AE patients. Consistently, the literature showed an increased risk of developing epilepsy in AE patients with a delay in the initiation of immunotherapy (4). This may be because immunotherapy delay is associated with an increased likelihood of aggravation of the autoimmune process in the brain, which leads to the development of epilepsy. Seizure freedom was reached soon after the initiation of immunotherapy, and AE patients receiving early immunotherapy were more likely to achieve seizure freedom faster (6). Thus, in clinical practice, we should prioritize immunotherapy to control acute seizures as soon as possible and improve seizure outcomes after the acute phase.

Chen et al. recently reported that among patients with anti-NMDAR encephalitis, those with brain MRI abnormalities were more likely to achieve seizure control than were patients in the normal MRI group, indicating a potential association between acute seizures and brain MRI results (23). However, the current study found that brain MRI results were not associated with acute seizure or the development of epilepsy in patients with anti-NMDAR, anti-LGI1 and anti-GABABR encephalitis, which is in agreement with prior studies (4, 8).

There are several limitations to this study. First, the sample size was relatively small, and all patients were enrolled from a tertiary hospital in Northeast China. Thus, selection bias may exist, and the sample may not be representative of all regions in China and other countries. Second, seizure outcomes were confirmed based on telephone interviews in some patients if they did not report epileptic seizures during follow-up, and recall bias may thus exist. Third, follow-up times were inconsistent and ranged from 6–60 months among patients. Some patients were followed-up for only 6 months, and a short follow-up time may affect the evaluation of seizure outcomes and lead to an underestimated incidence of epilepsy. Fourth, only patients with anti-NMDAR, anti-LGI1 and anti-GABABR encephalitis were included in our study. These three types of AE are the three most common neuronal surface antibody-mediated AEs. We excluded patients with other types of AE, which may limit the interpretation of the associations between seizure outcomes and the type of AE. Our findings may also not be relevant for all AE patient groups. Finally, the neurological outcome was not determined in AE patients during follow-up, and the possible association between neurological outcome and the development of epilepsy was not identified.

## Conclusion

In the acute phase of AE, acute symptomatic seizures were highly prevalent manifestations and were reported by 86% of patients. During a median follow-up of 21 months, 28.4% of the patients with acute seizures developed epilepsy. The independent factors that predicted the development of epilepsy after the acute phase included a larger number of ASMs, EEG abnormalities, and immunotherapy delay. In clinical practice, we should prioritize immunotherapy to control acute seizures as soon as possible and improve seizure outcomes after the acute phase. For AE patients with an increased risk of developing epilepsy, early withdrawal of ASMs is not recommended.

## Data Availability Statement

The raw data supporting the conclusions of this article will be made available by the authors, without undue reservation.

## Ethics Statement

The studies involving human participants were reviewed and approved by the medical ethical committees of Jilin University, First Hospital. Written informed consent from the participants’ legal guardian/next of kin was not required to participate in this study in accordance with the national legislation and the institutional requirements.

## Author Contributions

WL and RZ conceived of and designed the study. XZ, ML, and XG were involved in data acquisition. QC and RZ analyzed the data and wrote the manuscript. All authors contributed to the article and approved the submitted version.

## Conflict of Interest

The authors declare that the research was conducted in the absence of any commercial or financial relationships that could be construed as a potential conflict of interest.

## Publisher’s Note

All claims expressed in this article are solely those of the authors and do not necessarily represent those of their affiliated organizations, or those of the publisher, the editors and the reviewers. Any product that may be evaluated in this article, or claim that may be made by its manufacturer, is not guaranteed or endorsed by the publisher.
